# Suspected myocarditis in patients with COVID-19: A multicenter case series

**DOI:** 10.1097/MD.0000000000024552

**Published:** 2021-02-26

**Authors:** Natascia Laganà, Marco Cei, Isabella Evangelista, Scilla Cerutti, Alessandra Colombo, Lucia Conte, Enricomaria Mormina, Giuseppe Rotiroti, Antonio Giovanni Versace, Cesare Porta, Riccardo Capra, Valerio Vacirca, Josè Vitale, Antonino Mazzone, Nicola Mumoli

**Affiliations:** a Department of Internal Medicine, ASST Ovest Milanese, Magenta and Legnano (MI); b Department of Clinical and Experimental Medicine, Policlinico Universitario “G. Martino”, University of Messina, Messina; c Department of Internal Medicine, Cecina Hospital, Cecina (LI); d Biomedical and Dental Sciences and of Morphofunctional Imaging, University of Messina, Messina, Italy.

**Keywords:** COVID-19, myocarditis, pneumonia

## Abstract

Although myocarditis can be a severe cardiac complication of COVID-19 patients, few data are available in the literature about the incidence and clinical significance in patients affected by SARS-CoV-2. This study aims to describe the prevalence and the clinical features of suspected myocarditis in 3 cohorts of patients hospitalized for COVID-19. We retrospectively evaluated all the consecutive patients admitted for COVID-19 without exclusion criteria. Suspect myocarditis was defined according to current guidelines. Age, sex, in-hospital death, length of stay, comorbidities, serum cardiac markers, interleukin-6, electrocardiogram, echocardiogram, and therapy were recorded. Between March 4 to May 20, 2020, 1169 patients with COVID-19 were admitted in 3 Italian Medicine wards. 12 patients (1%) had suspected acute myocarditis; 5 (41.7%) were men, mean age was 76 (SD 11.34; median 78.5 years); length of stay was 38 days on average (SD 8, median value 37.5); 3 (25%) patients died. 8 (66.7%) had a history of cardiac disease; 7 (58.33%) patients had other comorbidities like diabetes, chronic obstructive pulmonary disease, or renal insufficiency. Myocarditis patients had no difference in sex prevalence, rate of death, comorbidities, elevations in serum cardiac markers as compared with patients without myocardial involvement. Otherwise, there was a significantly higher need for oxygen-support and a higher prevalence of cardiac disease in the myocarditis group. Patients with suspected myocarditis were older, had a higher frequency of previous cardiac disease, and significantly more prolonged hospitalization and a lower value of interleukin-6 than other COVID-19 patients. Further studies, specifically designed on this issue, are warranted.

## 1 Introduction

A novel beta-Coronavirus related to Severe Adult Distress Syndrome, firstly isolated in December 2019 in Wuhan, Central China^[1]^ and named SARS-CoV-2, is currently pandemic, with more than 24 million people contaminated. COVID-19, the disease provoked by the virus, resulted in more than 80,000 deaths in quite 200 countries worldwide.^[1]^

Most patients have respiratory symptoms as fever, cold, cough, shortness of breath. However different clinical manifestations can be observed, from asymptomatic cases to mild and no-specific symptoms or severe complications as acute respiratory distress and death.^[2]^ Several cardiovascular complications^[3–5]^ were reported in literature like arrhythmias, myocarditis, pericarditis, heart failure, myocardial ischemia, myocardial infarction, and Takotsubo syndrome.

To our knowledge several cases of myocarditis associated with SARS-CoV-2 have been reported so far;^[6–10]^ nevertheless, to date, there is a knowledge gap regarding the real prevalence of this cardiac involvement, its pathologic pathway and specific characteristics of patients who experiment this complication.^[11]^

Thus, we reported a case series of clinically suspected myocarditis in our 1169 cohort-population.

## 2 Methods

Three Hospitals (Magenta and Legnano Hospitals in Lombardy – an area of Italy severely affected by the infection – and Messina Hospital in Sicily, all in Italy) were involved in this retrospective multicenter cohort study. We evaluated all the consecutive patients admitted in our Internal Medicine wards for symptomatic SARS-CoV-2 infection confirmed by positive polymerase chain reaction testing of nasopharyngeal specimens. We did not contemplate any exclusion criteria. A trained team of physicians performed a retrospective review of the electronic health records to obtain data on a standardized data collection form. Demographic data, underlying comorbidities, symptoms and signs at presentation, complications, treatment, and outcomes were collected and evaluated. Patients were considered to have suspected myocarditis if they had at least one of the typical clinical presentations of myocarditis and at least 1 diagnostic criteria, accordingly with the 2013 European Society of Cardiology (ESC) position statement^[12]^ (Table 1). Routine laboratory evaluation included complete blood count with differential, basic metabolic panel, liver function tests, ferritin, C-reactive protein, lactate dehydrogenase, and creatine phosphokinase, in addition to high-sensitivity cardiac troponin T and D-dimer, interleukin-6 (IL-6) and N-terminal pro-brain natriuretic peptide (NT-pro-BNP). Main outcomes included overall inhospital mortality and length of stay. The ethical approval for this study and the need for written informed consent was not required due to the retrospective nature of the study and the ethical considerations of this research were conformed to the Declaration of Helsinki. The study was carried out and reported according to the Strengthening the Reporting of Observational Studies in Epidemiology (STROBE) guidelines for observational studies.^[13]^ Continuous variables are expressed as mean plus or minus the standard deviation (SD) or as median with minimum and maximum values when data did not have a normal distribution according to the Shapiro and Wilk test; categorical data are given as counts and percentages. The Student's *t* test or Mann–Whitney *U* test was used for groups’ comparisons of continuous variables, depending on the distributional properties of the data. The chi-squared test (or Fisher exact test) was used to compare proportions. All analyses were conducted with SPSS statistical software version 20.0.

**Table 1:** Criteria for suspected myocarditis according with 2013 European Society of Cardiology (ESC) position statement.

One of the following clinical presentation *and* one of the following diagnostic criteria	
1. Acute chest pain (pericarditis or pseudo-ischemic), *or*	1. ECG/Holter stress test features – New 12-lead ECG and/or Holter and/or stress testing abnormalities with any of the following: first to third degree atrioventricular (AV) block or bundle branch block, ST/T wave change (ST elevation or T wave inversion), sinus arrest, ventricular tachycardia or fibrillation, asystole, atrial fibrillation, significantly reduced R wave height, intraventricular conduction delay (widened QRS complex), abnormal Q waves, low voltage, frequent premature beats, or supraventricular tachycardia
2. New-onset (days up to 3 mo) or worsening of dyspnea at rest or exercise, and/or fatigue, with or without left and/or right HF signs, *or*	2. Elevated troponin T or troponin
3. Palpitation, and/or unexplained arrhythmia symptoms and/or syncope, and/or aborted sudden cardiac death, *or*	3. Functional and structural abnormalities on cardiac imaging (echocardiogram, angiogram, or CMR) – New, otherwise unexplained abnormality of LV and/or RV function (regional wall motion abnormality or global systolic or diastolic dysfunction); such abnormality may occur with or without one or more of the following: ventricular dilatation, increased ventricular wall thickness, pericardial effusion or intracavitary thrombus.
4. Unexplained cardiogenic shock	4. Tissue characterization by CMR – Late gadolinium enhancement (LGE) and/or findings consistent with edema in pattern suggestive of myocarditis

AV = atrioventricular, CMR = cardiac magnetic resonance, ECG = electrocardiogram, HF = heart failure, LGE = late gadolinium enhancement, LV = left ventricular, RV = right ventricular.

## 3 Results

Between March 4 to May 20, 2020, 1169 patients with COVID-19 were admitted. The mean age was 70.96 (ST 16.71; median value 74 years) and 48% of patients were male. The length of stay was 30.32 days on average (SD 15.9, median value 27.5) (Table 2).

**Table 2:** Characteristics in COVID-19 Patients (N = 1169). Baseline clinical and laboratory features of COVID-19 patients.

Characteristic	Value	Normal range
Female, n (%)	608 (52%)	–
Male, n (%)	561 (48%)	–
Age, mean (SD)	70.96 (16.71)	–
Length of stay, mean days (SD)	30.32 (15.9)	–
Death, n (%)	245 (21%)	–
Cardiac disease, n (%)	341 (29%)	–
Comorbidities, n (%)	620 (53%)	–
IL-6 level, mean (SD)	66.5 (47.37)	0–7 pg/mL
NT-proBNP, mean (SD)	1989 (5111.32)	0–125 pg/mL
T-troponine, mean (SD)	43.36 (30.17)	0–14 pg/mL
Oxygen-support, n (%)	327 (28%)	–

IL-6 = interleukin-6, NT-proBNP = N-terminal pro-brain natriuretic peptide.

Three hundred forty one (29%) patients had a history of cardiac disease. Among these patients, 136 (40%) had a history of heart failure, 85 (25%) had atrial fibrillation, and 119 (35%) had coronary artery disease (CAD), 204 (60%) had systemic hypertension. Six hundred twenty (53%) patients had other comorbidities like diabetes, chronic bronchitis obstructive disease, and chronic kidney disease. Two hundred nine (18%) had no cardiac disease or comorbidities.

During hospitalization, 245 (21%) patients died, 26 (2.3%) developed thrombo-embolic events, 222 (19%) acute respiratory distress syndrome, 33 (2.9%) septic shock, 35 (3%) atrial fibrillation, 2 (0.2%) Takotsubo syndrome, 140 (12%) bacterial sovra-infection of urinary tract and lung.

Twelve patients (1%) (Table 3) developed a clinically suspected acute myocarditis. Of these, 5 (41.7%) were men; the mean age was 76 (ST 11.34; median value 78.5 years). The length of stay was 38 days on average (SD 8, median value 37.5). 3 (25%) patients died. 8 patients (66.7%) had a history of previous cardiac disease. Among cardiac patients, 37.5% had a history of heart failure (2 patients with reduced ejection fraction 1 patient with mid-range ejection fraction), 50% had atrial fibrillation, 75% had CAD, and 75% had systemic hypertension. Two (25%) of these patients died. 7 (58.3%) patients had other comorbidities as previously defined. 5 (41.66%) patients had only cardiovascular diseases.

**Table 3:** Characteristics in Myocarditis Patients (N = 12). Baseline clinical and laboratory features of COVID-19 patients affected by myocarditis.

Characteristic	Value	Normal value
Female, n (%)	7 (58.3%)	–
Male, n (%)	5 (41.7%)	–
Age, mean (SD)	76 (11.34)	–
Length of stay, mean days (SD)	38 (8)	–
Death, n (%)	3 (25%)	–
Cardiac disease, n (%)	8 (66.7%)	–
Comorbidities, n (%)	7 (58.3%)	–
IL-6 level, mean (SD)	26 (8.5)	0–7 pg/mL
NT-proBNP, mean (SD)	1557.6 (2120.30)	0–125 pg/mL
T-troponine, mean (SD)	39.9 (37.94)	0–14 pg/mL
Day of diagnosis, mean (SD)	15.8 (10.5)	–
Oxygen-support, n (%)	9 (75%)	–

IL-6 = interleukin-6, NT-proBNP = N-terminal pro-brain natriuretic peptide, SD = standard deviation.

Interestingly, symptoms or signs of suspected acute myocarditis developed on the average 15.8 days of hospitalization (SD 10.52, median value 16); transthoracic echocardiogram (TE) demonstrated diffuse left ventricular hypokinesia, electrocardiogram (ECG) showed ischemic alteration in 8 patients, 3 QTc prolongation, 2 ECG were aspecific. Therapy with metil-prednisolone 1 mg/kg was started for all patients, 4 patients needed support with inotropic and vasoconstrictor drugs because of cardiogenic shock, 9 patients demonstrated a gradual recovery with improved TE findings after 6.75 days in average, 3 patients died. None of the patients who died was taking renin–angiotensin–aldosterone system inhibitors, the 9 patients who recovered were taking angiotensin-converting enzyme (ACE) inhibitors and angiotensin II receptor blockers (ARBs).

The incidence of myocarditis was 1%, with a lethal rate of 25% and a mortality rate of 0.2%. The 12 patients who experimented myocarditis at the admission had similar values of NT-proBNP, high sensitivity troponin T and IL-6 than the patients without myocarditis (Table 4). The 2 groups had no significatively differences in sex prevalence, rate of death, comorbidities (Tables 4 and 5).

**Table 4:** Comparison between the group of myocarditis (N = 12) and the group without myocarditis (N = 1157) of baseline clinical and laboratory features (*t* test).

	Normal range	Myocarditis Patients	No myocarditis Patients	*P* value
NT-proBNP, mean	0–125 pg/mL	1557.6	1907.7	*0.28*
IL-6, mean	0–7 pg/mL	26	65.7	*0.10*
Age, mean	–	76	71	*0.10*
Length of stay, mean days	–	38	29.5	***0.01***

IL-6 = interleukin-6, NT-proBNP = N-terminal pro-brain natriuretic peptide.

**Table 5:** Comparison between the group of myocarditis (N = 12) and the group without myocarditis (N = 1157) of baseline clinical and laboratory features (chi-square).

	Myocarditis patients (N = 12)	No myocarditis patients (N = 1157)	*P* value
Female, n (%)	7 (58.33%)	601 (52%)	*0.88*
Death, n (%)	3 (25%)	243 (21%)	*0.98*
Comorbidities, n (%)	7 (58.33%)	613 (52%)	*0.93*
Need of oxygen-support n (%)	9 (75%)	318 (27%)	***0.0009***
Cardiac disease, n (%)	8 (66.7%)	333 (28.8%)	***0.01***

There was a significantly higher need of oxygen-support (*P* value = .0009 chi-squared Yates-corrected), duration of hospitalization (*P* value = .01) and a significantly higher rate of cardiac disease (*P* value = .01 chi-squared Yates-corrected) in the myocarditis group (Tables 4 and 5).

Patients with comorbidities were older (with a trend of significance, *P* value .07), had significantly more prolonged hospitalization (*P* value .009) and a lower value of IL-6 (with a trend of significance, *P* value .09). They had the same values of NT-pro-BNP and high sensitivity troponin T than patients without comorbidities (Table 6).

**Table 6:** Myocarditis group: Comparison between patients with comorbidities (N = 7) and without comorbidities (N = 5) (*t* test).

	Normal range	Patients with comorbidities (N = 7)	Patients without comorbidities (N = 5)	*P* value
Age, mean (SD)	–	80.28 (12)	68.5 (2.12)	*0.07*
Length of stay, mean days	–	42.28 (5.7)	28.5 (9.2)	***0.009***
IL-6, mean (SD)	0–7 pg/mL	26 (8.5)	42 (18.4)	*0.09*
NT-proBNP, mean (SD)	0–125 pg/mL	1406.57 (1765.53)	3538 (4433.6)	*0.39*
T-troponine, mean (SD)	0–14 pg/mL	43.85 (50.5)	28 (8.5)	*0.34*

IL-6 = interleukin-6, NT-proBNP = N-terminal pro-brain natriuretic peptide, SD = standard deviation.

## 4 Discussion

The available evidence regarding the association between COVID-19 and myocardial injury showed that cardiac involvement resulted in a substantial proportion of infected patients.^[14]^ The first Chinese report showed that 12% to 28% of patients had elevated cardiac troponin levels.^[15]^ Available data reported that acute cardiac injury is more frequently observed in patients with more severe COVID-19 infections, and patients with cardiac injury are often aged subjects with comorbidities including hypertension, CAD and diabetes^[16]^; patients with a history of hypertension seem to be more exposed to cardiac damage.^[17]^

In the current literature, several mechanisms of cardiac involvement have been proposed: direct viral damage, ACE2-receptors down-regulation, adrenergic status, hypoxia, inflammatory response, drug toxicity.^[16]^ These mechanisms may result in different cardiovascular manifestations like arrhythmias, Takotsubo cardiomyopathy, heart failure, myocarditis, pericarditis, myocardial ischemia, myocardial infarction, microangiopathy with cardiac and peripheral involvement.^[18]^

Arrhythmia can be the result of underlying cardiac remodeling in pre-existent disease or the consequence of therapy with drugs such as azithromycin and hydroxychloroquine that can induce QT interval prolongation.^[19]^

Takotsubo cardiomyopathy in COVID-19 infection probably is triggered by both disease psychological stress and pro-inflammatory cytokine storm.^[20]^ Heart failure can result from the exacerbation of pre-existent cardiomyopathy.

Pericarditis is probably the consequence of pro-inflammatory cytokine storm in COVID-19 infection.^[11]^ Current data revealed high concentrations of interleukin-1 beta, interferon gamma, interferon inducible protein-10, interleukin-6 and monocyte chemoattractant protein-1 in patients with COVID-19 infection.^[21]^ This inflammatory damage mediated by overactivation of the immune system is suggested by sporadic autopsy cases showing infiltration of myocardium by interstitial mononuclear inflammatory cells^[22]^ and the patient's good response to glucocorticoid treatment.^[23]^

Pathogenesis of myocarditis is probably multifactorial, but to date, no evidence is demonstrating a predominant pathway in specific phenotypes COVID-19 patients; myocarditis can be the result of both direct viral damage and overactivation of the immune system, but current literature cannot define which phenotype COVID-19 patient has a major predisposition to the first or the latter pathogenic mechanism; moreover, other mechanisms such as adrenergic status and hypoxia due to pulmonary involvement are probably involved in myocarditis, as in heart failure and stress cardiomyopathy.^[20]^ These topics need to be investigated in more details, therefore we analyzed our COVID-19 population to define characteristics of myocarditis patients.

In our cohort, the incidence of suspected myocarditis was 1%, lower than the cardiac injury incidence reported in the literature.^[11]^ It is possible that myocarditis is currently underrated and misdiagnosed in COVID-19 infection clinical practice.

Patients who experimented myocarditis had no difference in rate of death, sex category, and extracardiac comorbidities. On the contrary, we noted a significatively higher prevalence of previous cardiac diseases. Myocarditis in our COVID-19 patients was associated with significant prolonged hospitalization and a higher need for oxygen-support in patients, but we are not able to discriminate if these were stigmata of more severe disease status or mere consequences of the cardiac complications: prospective studies are needed to clarify this issue.

In myocarditis patients, IL-6 levels were lower in patients with comorbidities than in those without comorbidities, but this result did not reach a statistical significance. We can speculate that this can be due to different pathogenic mechanisms underlying myocarditis: prevalent overactivation of the immune system in patients without comorbidities, who showed a higher level of IL-6 and who responded earlier to corticosteroid therapy; prevalent direct viral damage and pathologic changes to the renin-angiotensin-aldosterone system^[24]^ in patients with comorbidities, who were older, had a higher rate of cardiac diseases and needed longer support of inotropic therapy.

We recognize that our study has several limitations, first of all, the retrospective design and the small sample of patients with suspected myocarditis. In clinical practice, myocarditis represents a growing challenge for physicians, because it presents in many different ways, ranging from mild symptoms of chest pain and palpitations associated with transient ECG changes to life-threatening cardiogenic shock and ventricular arrhythmia.^[25]^ Viral myocarditis is mainly a diagnosis of exclusion, frequently supported by cardiac magnetic resonance imaging (CMR).^[12,25]^ Definitive diagnosis required endomyocardial biopsy (EMB).^[12,25]^ We did not perform cardiac CMR nor EMB in any patients because these diagnostic procedures are not universally available and the thinking that a more definite diagnosis probably would have not altered treatments, facing the risk of nosocomial spread of the virus.

## 5 Conclusion

Myocarditis is a severe cardiac complication in SARS-CoV-2 infection. Yet, no data are available in the literature about the incidence and clinical significance of myocarditis in patients affected by COVID-19. This study showed that in COVID-19 patients myocarditis was associated with more severe infection and a higher need for oxygen therapy, a higher rate of cardiac disease, and a longer hospitalization. Since myocarditis patients with comorbidities had counterintuitive lower levels of IL-6, this finding deserves to be further investigated to better understand myocarditis prognostic and pathogenetic factors and to evaluate a specific target therapy.

## Author contributions

N. Laganà, A. Mazzone, J. Vitale, M. Cei and N. Mumoli were responsible for the study concept and design; N. Laganà, I. Evangelista, G. Rotiroti, C. Porta, R. Capra, V. Vacirca, and N. Mumoli, were responsible for the acquisition of data; N. Mumoli, I. Evangelista, S. Cerutti, A. Colombo, L. Conte, J. Vitale, and M. Cei were responsible for the database handling and updating; N. Mumoli, N. Laganà and M. Cei were responsible for statistical analysis; N. Mumoli, N. Laganà, E. Mormina and AG. Versace were responsible for the drafting of the manuscript; N. Mumoli, N. Laganà and M. Cei were responsible for interpretation of results; N. Mumoli, N. Laganà, J. Vitale, M. Cei and A. Mazzone, were responsible for critical revision of the manuscript for important intellectual content. All authors had full access to the data and the accuracy of the data analysis.

**Conceptualization:** Natascia Laganà, Antonino Mazzone, Nicola Mumoli.

**Data curation:** Natascia Laganà, Marco Cei, Scilla Cerutti, Alessandra Colombo, Lucia Conte, Enricomaria Mormina, Giuseppe Rotiroti, Cesare Porta, Riccardo Capra, Valerio Vacirca, Antonio Giovanni Versace, Josè Vitale, Antonino Mazzone, Nicola Mumoli.

**Formal analysis:** Natascia Laganà, Alessandra Colombo, Lucia Conte, Enricomaria Mormina, Giuseppe Rotiroti, Cesare Porta, Riccardo Capra, Valerio Vacirca, Antonio Giovanni Versace, Josè Vitale, Antonino Mazzone, Nicola Mumoli.

**Investigation:** Natascia Laganà, Marco Cei, Isabella Evangelista, Scilla Cerutti, Alessandra Colombo, Lucia Conte, Enricomaria Mormina, Giuseppe Rotiroti, Cesare Porta, Riccardo Capra, Valerio Vacirca, Antonio Giovanni Versace, Josè Vitale, Antonino Mazzone, Nicola Mumoli.

**Methodology:** Natascia Laganà, Marco Cei, Scilla Cerutti, Josè Vitale, Antonino Mazzone, Nicola Mumoli.

**Resources:** Isabella Evangelista, Nicola Mumoli.

**Supervision:** Josè Vitale, Antonino Mazzone, Nicola Mumoli.

**Visualization:** Isabella Evangelista.

**Writing – original draft:** Natascia Laganà, Nicola Mumoli.

**Writing – review & editing:** Natascia Laganà, Marco Cei, Josè Vitale.
